# A systematic review of neural, cognitive, and clinical studies of anger and aggression

**DOI:** 10.1007/s12144-022-03143-6

**Published:** 2022-06-08

**Authors:** Yuliya Richard, Nadia Tazi, Dorota Frydecka, Mohamed S. Hamid, Ahmed A. Moustafa

**Affiliations:** 1 Blue Horizon Counseling Services, Sydney, Australia; 2 Arabian Gulf University, Manama, Bahrain; 3 Universite Med 5th, Rabat, Morocco; 4 Department of Psychiatry, Wroclaw Medical University, Pasteur Street 10, 50-367 Wroclaw, Poland; 5 College of Education, Ain Shams University, Cairo, Egypt; 6 Department of Human Anatomy and Physiology, the Faculty of Health Sciences, University of Johannesburg, Johannesburg, 2193 South Africa; 7 School of Psychology, Faculty of Society and Design, Bond University, Gold Coast, QLD Australia

**Keywords:** Neural studies, Anger, Violence, Aggression, Impulsivity, Anger management, Frontal cortex, Amygdala, Mindfulness, Cognitive behavioural therapy

## Abstract

Anger and aggression have large impact on people’s safety and the society at large. In order to provide an intervention to minimise aggressive behaviours, it is important to understand the neural and cognitive aspects of anger and aggression. In this systematic review, we investigate the cognitive and neural aspects of anger-related processes, including anger-related behaviours and anger reduction. Using this information, we then review prior existing methods on the treatment of anger-related disorders as well as anger management, including mindfulness and cognitive behavioural therapy. At the cognitive level, our review that anger is associated with excessive attention to anger-related stimuli and impulsivity. At the neural level, anger is associated with abnormal functioning of the amygdala and ventromedial prefrontal cortex. In conclusions, based on cognitive and neural studies, we here argue that mindfulness based cognitive behavioural therapy may be better at reducing anger and aggression than other behavioural treatments, such as cognitive behavioural therapy or mindfulness alone. We provide key information on future research work and best ways to manage anger and reduce aggression. Importantly, future research should investigate how anger related behaviours is acquired and how stress impacts the development of anger.

## Introduction

There are at least two views of emotions. First, emotions are perceived as discrete concepts (Ekman, 2016), comprised of six categories: anger, disgust, fear, happiness, sadness, and surprise. The discrete view of emotions has dominated psychology research for several decades, although there are arguments against it (Cowen et al., 2019). Cowen et al. (2019) argue that emotions are more complex than the basic six emotional categories. They argue that humans can experience a mix of these emotions, and also feelings that do not fall into these six categories. Further, another view is the social constructionist view, which considers emotions as products of brain processes in interaction with different social realities (Barrett, 2006). The latter view is more suited to better explain the complexities of emotional processes (including anger), and aligns well the high dimensional view of emotions by Cowen et al. (2019).

According to Blair (2012), anger occurs in different scenarios, including exposure to extreme heat, not receiving an expected reward, being treated unfairly, or actions by others that impact one’s goals or plans. Along these lines, anger feelings can occur when one’s goal is blocked (Berkowitz, 1993). For example, using simulated driving experiments, drivers may show anger feelings when they are forced to slow down (Stephens & Groeger, 2009). Another study found that sleep deprivation was related to the development of anger (Saghir et al., 2018). One study reported that anger and aggression can occur due to social rejection, frustration, provocation, and social stress (Lickley & Sebastian, 2018). Furthermore, state anger was shown to be associated with feelings of revenge (DiGiuseppe & Froh, 2002). Several studies have also shown that anger occurs due to social isolation and restrictions during the COVID-19 pandemic (Abadi et al., 2021; Aki et al., 2020; Smith et al., 2021).

Anger is important to investigate as it is an approach-, rather than avoidance- related response (Carver & Harmon-Jones, 2009). Anger is in contrast to other negative emotional feelings, such as depression or sadness, as the latter do not often lead to approach behaviours (Zhan et al., 2018). This has been experimentally corroborated using reaction time tasks of moving forward or backward in response to neutral or anger-related words (Mayan & Meiran, 2011). In this study, presenting participants with anger-related stimuli has led to moving forward more than when presenting them with neutral stimuli. This can possibly explain why anger can sometimes lead to aggressive behaviours.

Although important, anger-related disorders only appear in DSM, as symptoms of clinical disorders, such as oppositional defiant disorder and intermittent explosive disorder (APA, 2013). Many patient populations show problems with anger management (Lievaart et al., 2016), including borderline personality disorder (Critchfield et al., 2004). Several studies have found that anger is related to alcohol and drug abuse. One study found cannabis use disorder is associated with inability to control anger among Iraq and Afghanistan veterans (Dillon et al., 2021). Other studies also found that state and trait anger are risk factors for substance use and abuse (Baharvand & Malekshahi, 2019). Alcohol use disorders were found to be related to both state and trait anger (Sharma et al., 2017). The impact of alcohol on anger could be related to the activation of GABA receptors, and thus inhibiting the prefrontal cortex (Abernathy et al., 2010; Tu et al., 2007). As we discuss below, the prefrontal cortex plays a key role in anger control and a damage to this area may then increase anger-related behaviours.

The current paper also deals with few challenges in the literature, including the following: (a) what is the relationship between anger and aggression, (b) which cognitive processes are associated with anger and aggression, (c) what are the neural substrates of anger and aggressive behaviour, and (d) what are best interventions or counselling techniques for minimizing anger and aggression. Importantly, unlike prior work, here, we aim to link successful intervention to cognitive and neural substrates of anger and aggression.

Anger can often lead to aggression, which has negative impacts on the individual and society. While anger is an emotional feeling, aggression/violence is a behaviour that can occur mostly due to anger-related feelings. It is important to note that there are many differences between state and trait anger (for discussion, see Spielberger, 1988). According to Spielberger (1988), unlike trait anger, state anger is a transient subjective emotional feeling of intense fury and rage. We suggest that state anger is most likely initiated more by very extreme external factors, while individuals with high trait anger may show anger-related behaviours (e.g., violence, aggression, among others) in response to minor hostility, such as provocation or insult (Deffenbacher, 1992; Smith et al., 2004). While it is perceived as maladaptive, it has been argued that anger has a very important evolutionary value for personality building and growth (Williams, 2017). While it is often assumed that anger feelings will lead to aggressive behaviours (Cheriji et al., 2012), this is not always the case. This assumption is based on findings that anger feelings are very strong, making aggression a likely outcome. However, some anger management techniques were found to reduce anger but not aggression (Chambers et al., 2009), suggesting that anger and aggression are not always interrelated. Further, it is not clear if anger leads to either reactive (i.e., impulsive) or proactive (i.e., planned) aggression (Lickley & Sebastian, 2018). Unlike proactive aggression, impulsive aggression has been reported in many patient populations, such as schizophrenia and PTSD (Arseneault et al., 2000; Comai et al., 2012a, b; Hoptman, 2015).

**Fig. 1:**
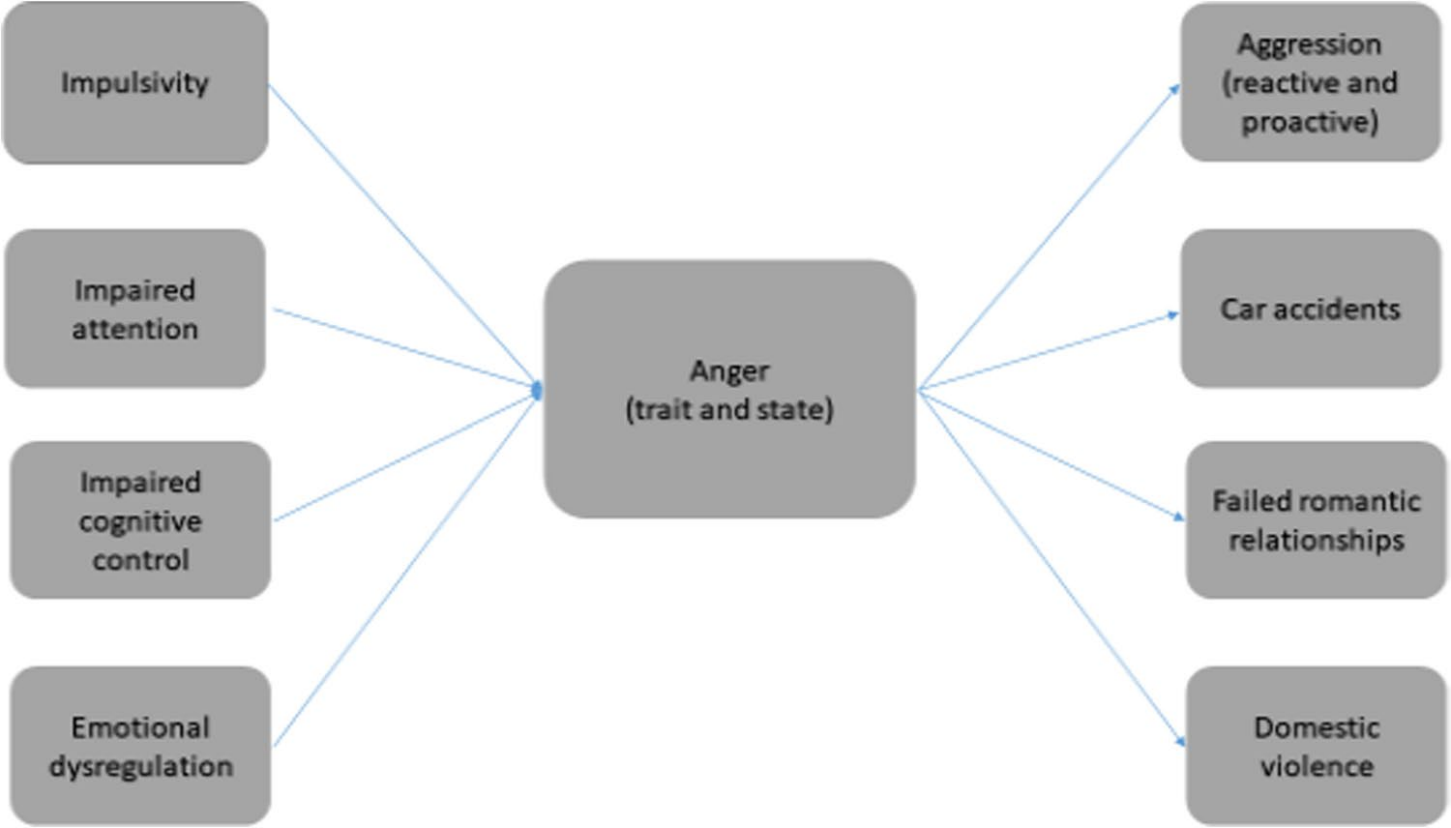
A description of cognitive processes underlying anger as well as consequents of anger

Anger is important to treat, as anger control deficits have negative consequences. A lack of anger control was found to negatively impact mental health (Prabhu et al., 2014) and lead to poor and maladaptive decisions (Meissner et al., 2021). For example, Masood et al. (2019) suggested that anger could be a factor underlying suicide ideation. Further, anger and suicide are were found to be common and related in younger than older adults (Khan & Hyder, 2006). Many individuals arrested for domestic violence incidents often undergo anger management training (Lee & DiGiuseppe, 2018), as anger is the likely culprit of violence-related behaviours. Further, anger can impact relationships and lead to domestic violence (Baron et al., 2007). See Fig. 1 for a description of negative consequences of anger.

Importantly, the goal of this article is to investigate the cognitive and neural substrates of anger and aggression, and then use this information to investigate best treatment for anger and aggression. It is hoped that effective treatments for anger should be able to ameliorate anger-related cognitive and neural dysfunction.

## Methods

**Fig. 2:**
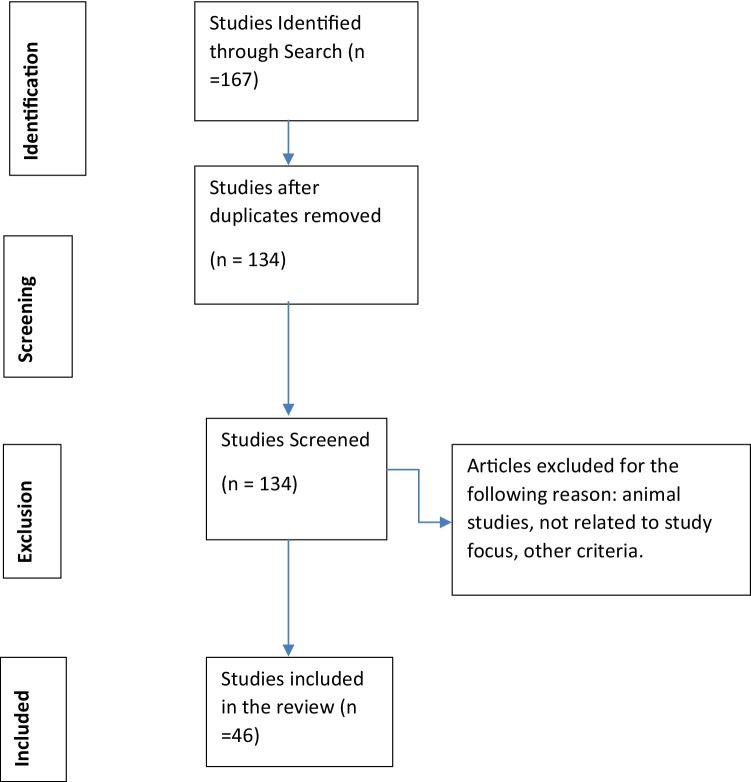
Search strategy used in our systematic review

In this systematic review, we have searched the following databases: Google Scholar, ProQuest, Pubmed, and PsychInfo. We have used a combination of two keywords in our searches. The first key word was anger, anger management, anger control, anger prevention, aggression, and anger-related processes. The second keyword was neural, brain, cognition, clinical, depression, schizophrenia, bipolar disorder, psychiatric (as well as variations of these words, such as bipolar). In addition, the search was limited to studies that used human participants and were published in English. Further, we have examined each paper carefully to make sure the goal of the study is examining anger and its relation to cognition, the brain, and clinical disorders. Studies that did not specifically measure anger were excluded. After removing duplicates and unrelated articles, here, we discuss 46 articles. Importantly, in discussion, we explain the relationship among all of these processes, such as the relationship between cognitive underpinnings of anger and its treatment. Please, see our search strategy in Fig. 2.

This study is designed in order to link successful interventions and counselling techniques for anger-related behaviours and aggression to existing cognitive and neural dysfunction. Accordingly, key words used in the systematic review were selected to first explain cognitive and neural abnormalities related to anger and aggression. Following that, key words related to intervention or counselling for anger and aggression are selected. After finding all related papers, we have divided our search findings into three related themes: cognitive correlates of anger, neural substrates of anger, and the treatment of anger. Below, we discuss each in detail, respectively.

## Cognitive Correlates of Anger: Focus on Attention and Impulsivity

Several studies have investigated cognitive processes underlying state and trait anger as well as aggression (Simmons et al., 2022; Wilkowski & Robinson, 2008, 2010). To our knowledge, at least, three studies found that anger is related to frontal-based cognitive processes, such as attention, cognitive control (Rueda et al., 2004; Zelazo & Cunningham, 2007) and emotional dysregulation (Koole & Tschacher, 2016; Szasz et al., 2011).

Studies have shown that anger is related to paying excessive attention to anger-related stimuli. For example, individuals high in trait anger pay more attention to anger- and hostile-related stimuli than individuals low in trait anger (Alia-Klein et al., 2018; Gable, et al., 2015a, 2015b). Some studies also have found that compared to individuals low in high trait anger, individuals high in trait anger tend to pay more attention to anger- and hostile-related stimuli in the environment (Wilkowski & Robinson, 2008). Using an emotional Stroop task that include anger-related stimuli, it was found that individuals who are high on trait anger show difficulty disengaging from anger-related stimuli (Eckhardt & Cohen, 1997; Putman et al., 2004; Smith & Waterman, 2005; van Honk et al., 2001). Using the visual search task, it was also found that individuals who are high on trait anger pay a lot more attention to anger-related than neutral stimuli (P. Smith & Waterman, 2003). Along these lines, it has been reported that trait anger impacts the functioning of brain regions responsible for attentional processes (Alia-Klein et al., 2018).

In addition to attention, several studies found that impulsivity is a key factor underlying the occurrence of anger-related processes. For example, Masood et al. (2019) investigated differences in impulsivity and anger in two groups of Pakistani students: suicide ideators and non-ideators. They found anger and impulsivity were higher than in suicide ideators than in suicide non-ideators (for similar results also see Daniel et al., 2009). Similar results on the association between impulsivity, anger, and suicide were reported in different Eastern and Western countries (Ammerman et al., 2015; McGirr et al., 2008; Wang et al., 2014). The anger-based Go/NoGo task was also used to measure impulsivity in relation to anger. In this task, participants were required to either respond (Go trials) or not to respond (NoGo trials) for different both neutral and anger-related stimuli. It was also found that trait anger is related to impulsivity in anger-based Go/NoGo task (Lievaart et al., 2018). See Fig. 1 for a description of cognitive processes underlying anger-related processes. In sum, most prior studies found that anger and aggression are related to paying attention to anger-related stimuli in the environment as well as impulsive behaviours.

## Neural Substrates of Anger

There are several brain areas that play a role in anger-related processes, including the amygdala and several frontal cortical areas. While there are other brain regions implicated in anger such as the hypothalamus (Gouveia et al., 2019) and the periaqueductal grey region (Blair, 2016; Gouveia et al., 2019), in this section, we will focus on brain regions related to understanding higher-level processes of anger and its treatment: the amygdala and prefrontal cortex.

Several studies show that the amygdala plays a role in anger processing (Alia-Klein et al., 2009, 2020; Blair, 2012; Carlson et al., 2010). For example, it has been shown that amygdala activation increases in response to the presentation of angry stimuli (Derntl et al., 2009). In patients with social phobia, the amygdala showed higher activation in response to fear stimuli (Stein et al., 2002). Amygdala lesion was found to impair ability to perceive fear and anger (Scott et al., 1997). It is argued that an increase in testosterone levels impacts amygdala activity, leading to anger induction (Batrinos, 2012). Importantly, the amygdala includes several subregions that belong to different functional systems (Moustafa et al., 2013; Swanson & Petrovich, 1998). One area of the amygdala is the medial and central nucleus of the amygdala, which plays a role in expression of emotions. Another area is the basolateral nuclei of the amygdala, which was found to play a role in emotional learning and threat detection (Silva et al., 2016). One study found that the medial amygdala plays a role in rivalry aggression while the central amygdala plays a role in predatory aggression (Haller, 2018). Another study reported that the central amygdala plays a role in maternal aggression (Bosch & Neumann, 2010). Interestingly, unlike the central nucleus, one study has reported that the basolateral amygdala plays a role in reactive aggression (Buades-Rotger et al., 2019). In sum, these studies show that the amygdala plays a key role in processing and encoding anger and aggression.

In addition to the amygdala, several frontal cortical areas play a role in anger-related processes. For example, it has been reported that the ventromedial prefrontal cortex (vmPFC) plays a key role in controlling anger (Alia-Klein et al., 2009; Klimecki et al., 2018) as well aggressive behaviours (Gilam et al., 2018; Yang et al., 2017). In one study, it was found that higher ventromedial prefrontal cortex activity is associated with experiencing less anger in the Ultimatum game (Gilam et al., 2015), suggesting that this brain area inhibit anger-related behaviours. In another study, it was found that the left anterior middle frontal gyrus (which is connected to the ventromedial prefrontal cortex) plays a role in anger control and reduction (Eshel et al., 2021). These findings are in agreement with studies showing frontal lesion or injury can lead to increased anger and aggression (Cristofori et al., 2016; Grafman et al., 1996; Seguin, 2009). In sum, prior studies show that the ventromedial prefrontal cortex plays a key role in anger control and reduction.

In addition to the ventromedial cortex and dorsal prefrontal cortex, other cortical regions, including the anterior cingulate and insula were found to play a role in anger and aggression, including reactive aggression (Denson et al., 2009; Kramer et al., 2007). An increase of activation in the anterior cingulate cortex and insula were reported in anger-inducing situations (Damasio et al., 2000).

**Fig. 3:**
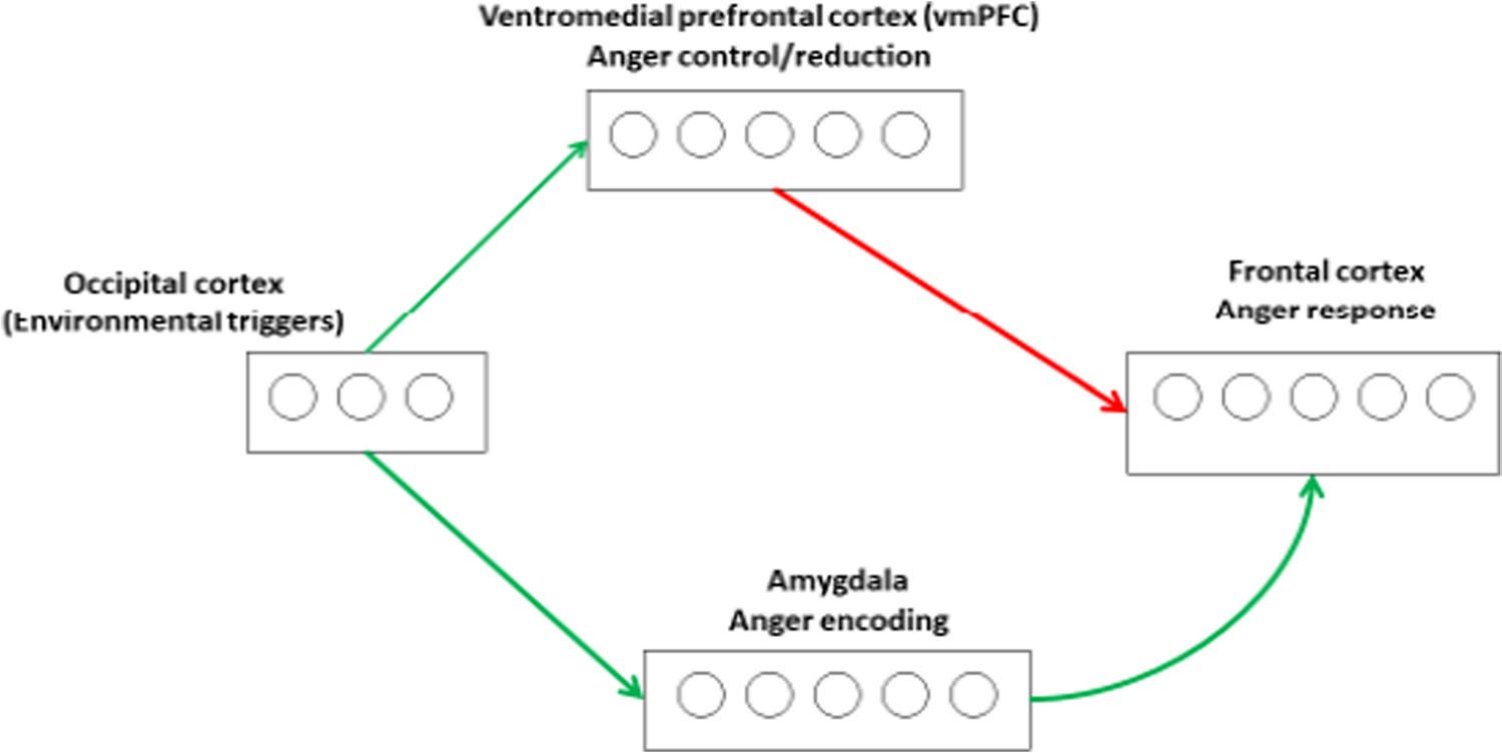
Neural underpinnings of anger, showing dual pathways of anger induction and control. While the amygdala plays a role in anger induction, the ventromedial prefrontal cortex and associated areas play a role in anger control. Green lines represent induction while red lines represent inhibition of anger responses. See text for discussion

It is important to note that these brain regions do not work in isolation, as the amygdala and other cortical areas discussed above are heavily interconnected. For example, it is known for several decades that frontal cortical areas, including ventromedial prefrontal cortex, insula, anterior cingulate, and dorsal prefrontal regions are connected via bidirectional pathways (Morawetz et al., 2016). Further, while the amygdala and ventromedial prefrontal cortex play a role in anger encoding and inhibition, they both projects to dorsal prefrontal regions responsible for the initiation of anger-related behaviours. This is supported by studies showing that the frontal cortex seems be the locus of anger-related behaviours, that is, anger expression (Blair, 2012). One study found that reactive aggression and anger is associated with a decreased connectivity between the amygdala and medial prefrontal cortex (Siep et al., 2019). Furthermore, most frontal cortical regions send projections to different subregions in the amygdala, including via the intercalated cells to the central nucleus as well as to the basolateral amygdala (Alexandra Kredlow et al., 2021; Ganella et al., 2017; Gold et al., 2016; Pare & Smith, 1993). However, it is not known how the interconnections among these cortical and subcortical structures mediate anger-related behaviours. Figure 3 shows a simplified neural network underlying anger encoding, expression, and reduction.

## Treatment of Anger and Aggression

There are different kinds of treatments for the treatment of anger and aggression. Cognitively oriented psychotherapies have been shown to be successful in reducing an individual’s tendency toward anger and reactive aggression (Beck & Fernandez, 1998; Deffenbacher et al., 2000). Anger management was found to decrease aggression (Valizadeh et al., 2010) as well as increase self-esteem (Bradbury & Clarke, 2007).

However, the most commonly used treatment for anger are cognitive behavioural therapy and mindfulness (see for example, Onwubiko, 2022). For a recent review on the use of cognitive behavioral therapy and mindfulness for the treatment of anger and aggression, see Didden et al. (2019). However, the Didden et al. (2019) review study did not compare which treatment, cognitive behavioural therapy or mindfulness, is more effective at treating anger and aggression.

Several studies have used cognitive behavioural therapy for the treatment of anger and aggression (Haustein et al., 2021; Lee & DiGiuseppe, 2018; Sukhodolsky et al., 2016). It has been found that cognitive behavioural therapy can effectively manage and reduce anger-related feelings and behaviours (Henwood et al., 2015). Cognitive behavioural therapies tend to modify erroneous beliefs (Meyerhoff & Rohan, 2016; Pittig et al., 2019). In terms of anger, some of the wrong beliefs include “I am a better driver than other drivers” (which can lead to anger feelings when a driver makes a minor mistake) or “I am a better partner than my wife” (which leads to anger feelings when a partner does minor mistakes). Further, several studies have reported that cognitive behavioural therapy is effective for the treatment aggression in patients with intellectual disabilities (Allan et al., 2001; Didden et al., 2016; Howells et al., 2000; Lindsay et al., 2003; Taylor & Novaco, 2013). Didden et al. (2016) argued cognitive behavioural therapy has some limitations (e.g., ineffective in some individuals as well as relapse) which need to be augmented with other treatment to address mental health problems in individuals who present with aggression problems.

In addition to cognitive behavioural therapy, mindfulness-based therapy is commonly used to treat anger and aggression. Mindfulness was found to be negatively related to anger and aggressive behaviour among drivers (Borders et al., 2010; Stephens et al., 2018). Mindfulness training is successful at reducing anger (Amutio et al., 2014; Fix & Fix, 2013; Heppner et al., 2008; Wright et al., 2009). Mindfulness helps to increase awareness to the current situation in order to assess it and act in a more appropriate way. Mindfulness training techniques were also found to decrease amygdala activity (Murakami et al., 2015), explaining perhaps how mindfulness is effective at treating anger. Mindfulness training includes training clients to disengage their attention from anger-related triggers (Didden et al., 2019). Several other studies have shown that mindfulness training successfully reduced aggression in individuals with intellectual disabilities (Singh et al., 2013), although it is not clear if these findings are generalizable to other populations.

Importantly, several studies have tested whether mindfulness based cognitive behavioural therapy is effective at treating anger and aggression (see for example, Kelly, 2007; Sohn et al., 2018). One study found that mindfulness based cognitive behavioural therapy is more effective at reducing anger in male taxi drivers than cognitive behavioural therapy (Kazemeini et al., 2013). Along these lines, it has been reported that mindfulness based cognitive behavioural therapy can effectively decrease impulsive behaviours and increase emotional regulation, thus managing anger and aggression (Clark, 2020). In a recent study conducted in Iran, it was found that mindfulness based cognitive behavioural therapy effectively improved anger control in males (Badpa et al., 2019). Mindfulness based cognitive behavioural therapy was also found to reduce driving anger (Diebold, 2003).

## Conclusion

In this review, we discussed the cognitive correlates of anger, neural substrates of anger, anger-related disorders, and the treatment of anger and its related disorders. In terms of cognitive underpinnings of anger, we found that impulsivity and impaired attention are related to anger processes.

Importantly, we found that like anxiety and drug seeking processes, there are different neural substrates for anger induction and anger control. The role of the ventromedial prefrontal cortex in anger control is similar to its role in reducing drug-seeking behaviours (Ebrahimi et al., 2019; Ghazizadeh et al., 2012; Konova et al., 2019; Peters et al., 2013; Radell et al., 2020; Sheynin et al., 2016) and anxiety (Hennings et al., 2020; Kalisch et al., 2006; Moustafa et al., 2013; Quirk et al., 2000; Radell et al., 2017; Scharfenort & Lonsdorf, 2016; Sierra-Mercado et al., 2010). These studies suggest that the ventromedial prefrontal cortex is very likely a control/inhibition mechanism for different behaviours, including drug seeking, anxiety, and anger This view is supported by recent studies on the role of vmPFC in anger and anxiety (Suzuki & Tanaka, 2021) and also on the close connection between anxiety and anger (Carre et al., 2012).

In short, the brain has likely evolved a mechanism that include regions for anger expression and different regions for anger reduction. This is most likely similar to other processes, such as the direct and indirect pathways in the basal ganglia that initiate and inhibit movement (Frank et al., 2007; Mandali et al., 2015; Moustafa et al., 2016), brain stimuli nuclei that control sleep (Hassani, Lee, & Jones), prefrontal-hippocampal circuit that control memory retrieval and submission (Benoit & Anderson, 2012), and amygdala regions that regulate fear initiation and expression (Strobel et al., 2015). For discussion on this topic, see Moustafa (2015).

Based on studies discussed above showing that anxiety and anger share some similarities, it is suggested that similar treatment strategies can be used for the treatment of both disorders (Brondolo et al., 1997). For example, cue exposure therapy has been extensively used for the treatment for anxiety (Suveg et al., 2018; Tay et al., 2019). Accordingly, Brondolo et al. (1997) suggested that cue exposure therapy can be used for the treatment of anger-related disorders. They suggested that like anxiety, anger can be triggered by some stimuli in the environment, and cue exposure therapy can teach patients with anger-related disorders to reduce their anger behaviours in relation to these triggers. Cue exposure therapy is an established treatment for anxiety disorders (Bahi & Dreyer, 2020; Javanbakht, 2018; Loucks et al., 2019; Nonkes et al., 2012; Stenmark et al., 2013). Other studies have also shown that cue exposure therapy can effectively decrease anger feelings (Stapleton et al., 2006).

Importantly, our review shows that mindfulness based cognitive behavioural therapy is more effective at treating anger and aggression than other behavioural treatments. This is possibly due to mindfulness based cognitive behavioural therapy ameliorates cognitive and neural abnormalities related to anger. For example, several studies found that mindfulness can increase attention and decrease impulsivity (Franco et al., 2016; Korponay et al., 2019; Liu et al., 2021; Wimmer et al., 2020) and also ameliorate ventromedial prefrontal cortex function (Kirk et al., 2014). Similarly, cognitive behavioural therapy was found to increase activity of cortical and subcortical structures impacted by anger, including prefrontal cortex, insula, and anterior cingulate (Porto et al., 2009; Seminowicz et al., 2013; Straube et al., 2006). These findings could explain why combining both mindfulness and cognitive behavioural therapy is more effective at managing anger than each therapy alone.

## Future Work

Given the similarity between anxiety, drug addiction, and anger (see above, and also see Walsh et al., 2018), future research should investigate environmental cues that leads to anger. Furthermore, there has been research on the acquisition of emotional responses in the domain of fear and anxiety. Along these lines, given the potential similar role for ventromedial prefrontal cortex and also amygdala in both anxiety and anger, it is possible treatments used for anxiety can successfully also manage anger. Our study has implications for counselling services in high (secondary) schools for mitigating violence and aggression (Arfasa & Weldmeskel, 2020). Counselling techniques should include methods to enhance anger control (by activating the ventromedial prefrontal cortex). These techniques can help improve academic and emotional performance of high school students (Arfasa & Weldmeskel, 2020). These points should be investigated in future studies.

Research has shown that anxiety can be acquired (Barot et al., 2009; Park et al., 2020; Rio-Alamos et al., 2015). However, to our knowledge, there are almost no research studies on how anger responses are acquired (or learned) in animals and humans. One exception is a study by Stephens and Groeger (2011) showing anger elicited in one situation can be carried over to subsequent similar scenarios.

Further, anger and aggression have been defined across different dimensions, such that there are state and trait anger and reactive and proactive aggression (see Introduction). However, it is not known how the different types of anger to relate to different types of aggression, which should be investigated in future work.

Furthermore, it is not clear why people are easier to have anger feelings when distressed (O'Grady et al., 2012; Onyedibe et al., 2020). It is possible that this is due to distress increases amygdala activity (Chen et al., 2017; Ressler, 2010), leading to anger induction. Another potential mechanism is stress causes homeostatic imbalance (Ladakis & Chouvarda, 2021), which can, in turn, increase anger feelings and expression (Robins & Novaco, 1999; Sorci et al., 2013; Williams, 2017). Further, future work should investigate mediating factors underlying the distress-anger relationship. It is possible that emotional regulation and mindfulness can help reduce anger in distress-related situations.

While it is well-documented that anger may impact decision making and lead to impulsivity, the reverse could also be correct. In other words, an individual’s decision making style may relate to their anger feelings. This can be studied in future studies by using the General Decision Making Style Questionnaire (Scott & Bruce, 1995). Individuals who score low in the rational decision making questions may also show more anger feelings.

Importantly, future research should also compare the existing treatments of anger and aggression including mindfulness and cognitive behavioral therapy. To our knowledge, there is only study that have compared mindfulness and cognitive behavioural therapy in the context of driving (Kazemeini et al., 2013). Given prior studies and the link between anger and distress, impulsivity, and emotional dysregulation, it is likely that mindfulness will be more effective at managing anger. Further, future work should investigate how both cognitive behavioural therapies and mindfulness modify erroneous beliefs related to anger, as discussed above (Meyerhoff & Rohan, 2016; Pittig et al., 2019).

## Data Availability

No data is collected in this systematic review.
